# Health shocks and labour market outcomes: evidence from The Irish Longitudinal Study on Ageing (TILDA)

**DOI:** 10.1186/s13561-026-00742-y

**Published:** 2026-02-25

**Authors:** Likun Mao, Charles Normand

**Affiliations:** 1 Department of Economics, University of Aberdeen, Aberdeen, AB24 5FX United Kingdom; 2 The Irish Longitudinal Study on Ageing, Trinity College Dublin, Dublin, D02 R590 Republic of Ireland; 3 Centre for Health Policy and Management, Trinity College Dublin, University of Dublin, 2-4 Foster Place, Dublin, D2 Republic of Ireland; 4 Cicely Saunders Institute, Kings College London, Bessemer Road, London, SE5 9PJ United Kingdom

**Keywords:** Health shocks, Labour supply, Healthcare entitlements, Ireland

## Abstract

**Background:**

Population ageing and later pension ages make older adults’ ability to work after adverse health events a key policy issue, yet causal evidence for Ireland is scarce. We use the Irish Longitudinal Study on Ageing (TILDA) to estimate the causal impact of unanticipated health shocks on labour-market outcomes during the post-crisis period 2009–2018.

**Methods:**

The sample includes 1,885 individuals aged 50–65 who were in paid work at baseline and observed for up to four biennial waves (4,160 year-to-person observations). Health shocks are analysed and defined as: (i) recent hospitalisation and (ii) first diagnosis of a chronic condition. To address selection into health events we apply inverse probability weighting with regression adjustment (IPWRA), which is doubly robust to misspecification of either the treatment or outcome model. This paper further discusses the role of public healthcare entitlements (GP/Medical card holder) as an important institutional dimension. Heterogeneity is explored by demographics and socioeconomic status. Various employment and health outcomes are examined.

**Results:**

A new chronic condition lowers the probability of remaining employed in the following wave by 3.0 percentage points (≈ 16% increase in the exit rate); hospitalisation reduces annual wages by 3,600 euros but shows no significant effect on hours worked. Both shocks predict deteriorating health conditions and higher healthcare utilisation. Employment effects are larger for women, low-SES respondents, and individuals outside the Dublin region. Possession of a public health entitlement raises the short-term risk of job exit yet, conditional on staying employed, appears to protect working capacity.

**Conclusions:**

For Irish older workers in the 2010s, acute health shocks reduced labour supply weakly on average, but the burden is concentrated among socio-economically disadvantaged groups. Universal access to subsidised primary care appears to cushion employment losses. Policies that expand early access to public health entitlements could therefore support longer working lives without exacerbating inequality.

**Supplementary Information:**

The online version contains supplementary material available at 10.1186/s13561-026-00742-y.

## Introduction

Increased life expectancy and low fertility are increasing the old-age-dependency ratio in all OECD countries, increasing upward pressure on social welfare programmes. Therefore, it is important to consider factors potentially limiting labour force supply of older workers. Health is an important dimension as age-related health issues emerge among older workers and play an influential role, reducing well-being at an individual level and incurring social costs.

There are well-established studies of the effects of health on labour market outcomes, generally confirming that poorer health is associated with negative labour market outcomes and highlighting the bidirectional nature of the health-work relationship [1–3]. The expected link can be illustrated using the health production model [4]. Evidence from Europe and the United States shows an increased likelihood of labour market exit associated with an ill-health status [5, 6]. Conversely, individuals with impaired health tend to experience longer unemployment spells [7], and a large body of research documents adverse health effects of labour market difficulties such as unemployment and economic uncertainties [8–11]. Given the reciprocal relationship, a growing literature investigates the effect of incident health events rather than general health status on labour market transitions, exploiting exogenous shocks or within-individual variation to account for persistent individual-level unobservables [12–15]. Acute shocks such as cancer, heart attack, and stroke can substantially increase the risk of older workers leaving the labour market [12]. Health shocks are also associated with negative economic consequences in both the short and long term [13, 16].

The literature on health shocks identifies several issues. The first concerns heterogeneities. Different sorts of health shocks lead to different responses in terms of treatments and consequences. For example, Smith (2005) [13] draws data from the Health and Retirement Survey (HRS) and shows that the short-term effect of a major health event is around a 15 percentage points (pp) decline in the probability of working, and less than 5 pp for a minor one. Different institutional and cultural environments partially explain the varying magnitude of the health effect across countries. In Europe, it is found that the employment effect is larger in Ireland, Netherlands, Denmark and Spain but is relatively smaller in a highly regulated labour market such as Italy [5, 6, 12, 15]. By comparison, in the U.S. where the health care system is characterised by more private funding, the economic costs of an adverse health event can be more serious. For instance, the recent study by Mommaerts et al. [17] combines the Survey of Health, Ageing and Retirement in Europe (SHARE), HRS and China Health and Retirement Longitudinal Study (CHARLS) to estimate the effect of hospitalisation on a range of financial consequences such as out-of-pocket (OOP) expenses, bankruptcy and income, and demonstrates apparent different patterns across countries^1^.

The second consideration is the time span between a health shock and subsequent outcomes, which has important implications in terms of behavioural adaptation and labour market rigidity. This also poses challenges in modelling the dynamic features of labour market transitions given empirical evidence on the multi-stage retirement among older people [18–21]. Limited by data availability, especially when taking account of previous individual conditions as controls for endogeneity, most studies have focused on a one or two-year period. Some recent studies investigate a longer period (mostly based on a general working population) and find a persistent negative effect, especially on the household income [16, 22, 23].

Finally, the underlying mechanisms are complex and remain less explored. Previous studies usually approach this issue by investigating the heterogeneous effects in subgroups by demographic features, or by the level of impairment, and socioeconomic status (SES). Garcia-Gomez (2011) [15] discusses the role of institutional arrangements in explaining heterogenous adjustments to a health shock across European countries. Trevisan and Zantomio (2016) [12] evaluate a series of shock-related outcomes that are potentially related to the adjustment of labour supply such as mental health and life expectancy. They suggest the role of subjective desire as well as families and disability benefits in determining the routing of labour market exit. Further investigation of the underlying mechanisms will help inform policymakers and help in the design of targets and labour supply incentives.

In the Irish context, there is not much evidence evaluating the effects of health on labour market participation (LMP) in older people using large-scale survey data. Some earlier studies investigate ill-health related retirement in some occupations [24, 25]. Gannon (2005) [26] investigates the effect of disability on LMP using the European Community Household Panel Survey (ECHPS) 1995–2000. By employing a range of panel models that accommodate state dependence and heterogeneity, the author shows that the unobserved heterogeneity contributes substantially to the base effect of disability for men. Disabled men with a current severe limitation are 9 pp less likely to participate compared to non-disabled men. In another work by Gannon and Roberts (2011) [27], the health effect is evaluated in terms of the retirement patterns such as a partial retirement. Using the Living in Ireland (LII) survey 1995–2001, they estimate the effect of having a health limitation on the LMP and find an increase in the probability of retirement, an average marginal effect of around 15 pp. But an insignificant effect is found for the part-time work to retirement. More specific to health events, there is only one relevant pan-European study based on ECHPS. Garcia-Gomez (2011) [15] finds a larger drop in the probability of being employed in Ireland compared to other countries: among those aged between 50 and 64, experiencing a health event defined as a change in chronic condition or self-assessed health (SAH) reduces the probability of staying employed by 10–16 pp. Such an effect is almost twice larger compared to many other European countries, which is suggested to be explained by the low integration of disabled people under current Irish disability policy^2^.

In the spirit of previous research, this paper contributes to the current literature on health shocks and labour market outcomes in two ways. First, it provides contemporary evidence on the causal effect of health shocks on labour market participation using an Irish working cohort after the financial crisis in 2009. Previous European studies predominantly rely on pre-2010 data^3^, potentially missing behavioural shifts influenced by economic instability, diminished employment opportunities, and changing work-related benefits. The financial sector collapse, bursting of the property bubbles, and a ballooning government deficit made Ireland one of the most severe countries hit by the crisis: for instance, GDP fell by 11.3% in 2009, while the public deficit increased to 14.3% of GDP, the largest among OECD countries^4^ [28]. In this altered economic landscape after the peak of the Celtic Tiger years (1995–2007), individuals may have remained in employment despite poor health to avoid financial hardship. Moreover, re-entry after a health event could be more difficult during downturns when vacancies are scarce.

To date, there is limited research exploring the causal relationship between health shocks and labour participation post-recession. The only known work uses the English Longitudinal Study of Ageing (ELSA) to investigate the labour supply response to an acute health shock and suggests a substantial increase in the baseline probability of labour market exit along with reduced hours and earnings [29]. Given recent economic disruptions, including those arising from the COVID-19 pandemic, this study helps shed light upon people’s resilience or vulnerability to health shocks in a particular financial environment.

Second, we highlight the role of Ireland’s institutional setting in shaping heterogenous labour market responses to health shocks. Earlier studies drawing on pan-European datasets primarily discuss labour market features and disability benefits in explaining cross-country heterogeneities [12, 15]. Comparing many other European countries, two features of Irish healthcare system are particularly salient for work adjustments after a health event. First, during our study period (2009–2018), Ireland did not operate a statutory sick pay scheme, so income protection during short-term illness depended largely on employer policies and the state benefit system (e.g., illness benefit)^5^. Second, Ireland does not provide universal coverage of primary care and has relatively high direct OOP expenditure to finance general practitioner (GP) care [32]. Emerging Irish research has also documented how variations in healthcare entitlements and GP accessibility influence healthcare utilization patterns [33, 34]. Together, these features may make individual’s decisions after health shock more sensitive to financial constraints. In this paper, we disentangle within-country institutional differences and investigate the role of healthcare entitlements in people’s follow-up treatments with regards to their earlier health event.

Our analysis is based on a nationally representative dataset, the Irish Longitudinal Study on Ageing (TILDA). Aiming to identify a causal relationship between health shocks and labour market outcomes, we employ a propensity-based approach, inverse probability weighting regression adjustment (IPWRA), to deal with potential endogeneities, especially selection bias. Different to earlier research, we find a negative but weak effect of experiencing a health event, either hospitalisation or the onset of a chronic condition, on labour market outcomes of those between age 50 and 65. Our estimates demonstrate around a three per cent lower likelihood of keeping an active working status in response to a health event in the short term within two years. The identified health shocks are found to be correlated with worse health conditions but seemingly not rising financial difficulties in the short or medium term.

The remainder of the paper is organized as follows. Sections 2 and 3 introduce our identification strategy and data. Section 4 presents our main results about the effect of health shock on labour market outcomes, and discussions on healthcare entitlements. Then it is followed by several robustness checks. We conclude in the final section.

## Empirical strategy

Conceptually, we could outline a simple estimation as follows:

$$\:{Y}_{it}=\alpha\:+\theta\:{D}_{it}+\beta\:{X}_{it}+{\epsilon\:}_{it}$$

Where $\:{Y}_{it}$ is labour market outcome variables for individual *i* at the time *t*. $\:{D}_{it}$ is an indicator for a health event.$\:\:\:{X}_{it}$ is a set of individual characteristics.

Identifying the health effect $\:\theta\:$ on labour market outcomes poses several challenges in a non-experimental study. First, omitted variables might confound the relationship between people’s labour market decisions and health status. For instance, people in some occupations are more likely to be exposed to work injuries that further transit to future impairments. Other unobserved characteristics such as personal traits might also affect both health behaviours and occupational choices, causing omitted variable bias.

Second, a reverse causality between health and labour market status would potentially bias the estimates especially for a multi-dimensional concept of health. For instance, current research still lacks conclusive results on the impact of retirement on health. Some find a negative impact on cognitive function [35, 36] or chronic conditions [37] whilst retirement is found to be associated with improved subjective health, reduced hospitalisation, and better health behaviours [38–40]. A presumably positive relationship between retirement and health would upward bias the estimate of the impact of health on LMP. Furthermore, older people might adjust their labour market activities even before experiencing a health shock according to their expectations for both employment and health status, which might be more apparent when they are approaching the effective retirement age.

Third, a measurement issue exists in multi-dimensional health status of which proxies have been used in different methods in existing literature. Subjective measure such as self-assessed health (SAH) is used to provide a comprehensive measure of health status, which however is subject to potential endogenous reporting, and the measurement error can be systematically related to labour market status [41]. For instance, those who are inactive may have an incentive to report worse than actual health to justify their inactivity, which is known as ‘justification bias’ and is more prominent when it comes to people near retirement age [42]. Therefore, more objective measures for health conditions or event-based measures such as diagnosis and hospitalisation help reduce reporting bias [41, 43].

Bearing these in mind, recent research has employed fixed effects models, difference-in-difference, or propensity score matching (PSM) to identify a plausible causal relationship between health and labour market activities. There is also some research based on the event study approach [14, 16]. In this paper, we use a double robust estimation using inverse propensity weights regression adjustment (IPWRA) to address the counterfactual problem present in observational data.

Formally, under the general framework of potential outcome models (POMs) [44–46], the outcome variable of interest $\:{Y}_{i}$ can be $\:{Y}_{1i}$ or $\:{Y}_{0i}$ depending on a different treatment status ($\:{D}_{i}=1\:or\:{D}_{i}=0)$. A missing data issue arises as we can not observe $\:{Y}_{1i}$ and $\:{Y}_{0i}$ at the same time for one individual so that we can only observe $\:{Y}_{i}={Y}_{0i}+({Y}_{1i}-{Y}_{0i}){D}_{i}$. Estimating the difference-in-means (unadjusted regression) would be:

$$\:E\left[{Y}_{1i}-{Y}_{0i}\right]=E\left[{Y}_{1i}|{D}_{i}=1\right]-E\left[{Y}_{0i}|{D}_{i}=0\right]=E\left[{Y}_{1i}-{Y}_{0i}|{D}_{i}=1\right]+E\left[{Y}_{0i}|{D}_{i}=1\right]-E\left[{Y}_{0i}|{D}_{i}=0\right]\:\:$$

This does not identify the average treatment effect (ATE) that comprises an average treatment effect on the treated (ATT) $\:E\left[{Y}_{1i}-{Y}_{0i}|{D}_{i}=1\right]$ and a selection bias term $\:E\left[{Y}_{0i}|{D}_{i}=1\right]-E\left[{Y}_{0i}|{D}_{i}=0\right]$. The bias term goes to zero under conditional unconfoundedness and overlap. Then, we can recover our treatment effect as ATT.

IPWRA method takes this selection bias into consideration by combining a weighting strategy and a regression adjustment method. It is primarily built on the regression adjustment (RA) that uses a difference in the average predictions for the treated and the average predictions for the nontreated. The RA estimator identifies the average treatment effect by comparing the mean of the predicted working status via separate multivariate regression for the treated and control groups, which ultimately compares two main POMs for the two groups. The ATT can be further calculated by comparing such a difference only among those treated. Furthermore, the inverse probabilities of treatment are applied to estimated coefficients, which requires a first stage of estimating the propensity of experiencing a health shock. Heavier weights would be imposed on observations that occur in the range where the distribution of the propensity is sparser. So, the inverse propensity weights magnify treatment individuals who otherwise look like they would not have the treatment and magnify control individuals who otherwise look like they would have selected treatment. If individuals were randomly selected into health shocks, the probabilities would be equal, and the estimator is equivalent to unweighted least squares. Formally, the IPRWA estimator could be written as:

$$\:{{\updelta\:}}_{ATT-IPRWA}=\frac{1}{{N}_{T}}\sum\:_{i=1}^{{N}_{T}}\left[{(\widehat{\alpha\:}}_{1}+{\widehat{\beta\:}}_{1}{X}_{i}\right)-({\widehat{\alpha\:}}_{0}+{\widehat{\beta\:}}_{0}{X}_{i})]$$

Where the $\:{(\widehat{\alpha\:}}_{1},{\widehat{\beta\:}}_{1}){\:and\:(\widehat{\alpha\:}}_{0},{\widehat{\beta\:}}_{0})$ are the estimated coefficients from the step of estimating separate probability-weighted linear outcome equations for the treated and control groups; $\:{N}_{T}$ is the number of treated observations [47, 48].

IPRWA is selected as our main strategy for two main reasons. For one thing, IPRWA improves PSM in terms of efficiency because including control variables in treatment equations would absorb variance more compared to comparing the direct difference between outcomes in the randomly selected treatment and control groups. In contrast to PSM which usually compares only one or a few observations around a propensity point, IPRWA considers all observations by more diversified weights. For another, IPRWA has a double robust property [47, 49], a remarkable property of accommodating potential misspecifications in either propensity model or the regression model predicting the outcomes.

This paper estimates the effect of a health event occurring between wave t and t + 1 on labour market outcomes measure at t + 1. Following growing literature on health shock [15–17, 23], we examine two incident health shocks: (1) first hospitalisation/Emergency Department (ED) use since baseline wave (2) first diagnosis of a chronic condition since the baseline wave (more details are provided in the Data section). These events occurred before the observed labour market status in the next wave, and are salient and time-stamped, reducing reverse causality and reporting concerns. However, it should be acknowledged that diagnosis measure may be susceptible to detection bias and hospitalisation reflects admission thresholds that may vary across providers and healthcare entitlements. The main measures are also limited in reflecting severity of the health shock, limiting further estimate on the extensive effect. We therefore complement additional analysis using limiting conditions and found consistent results.

IPWRA identifies the ATE by combining a treatment model for shock incidence with an outcome regression; consistency holds if either is correctly specified. We mitigate reverse causality by focusing on incident events and measuring outcomes at the subsequent wave, and by conditioning on rich pre-event covariates (demographics, socioeconomic status, job characteristics and health status). IPRWA relies on an unconfoundedness assumption conditional on a set of observed characteristics $\:{X}_{i}$ (also called conditional independence assumption (CIA)) to achieve independence between treatment status and the potential outcome. Propensity-based method requires an overlap assumption so that each has a positive probability of receiving treatment to ensure comparable controls for some treated observations. We conduct corresponding robustness checks over these assumptions in the following section.

## Data

All data examined by this paper are from the Irish Longitudinal Study on Ageing (TILDA), a nationally representative sample of community-dwelling individuals aged 50 years and over, and their spouses or partners at any age. This dataset is harmonised with many other international longitudinal datasets about ageing such as the US Health and Retirement Study (HRS), the Survey of Health, Ageing and Retirement in Europe (SHARE), and the English Longitudinal Study of Ageing (ELSA). The first wave covers the period from October 2009 to February 2011, and the follow-up waves take place approximately every two years. 8,504 individuals were sampled in the first wave. For our purpose, our analytical sample includes respondents aged 50–65 (i.e., below state pension age of 66) who are observed in at least two consecutive waves across the five TILDA waves (2009–2018). Our sample also excludes proxied interviews, people living in a nursing home^6^, or reporting an inconsistent labour market status. To limit undue influence from a small number of extreme observations and to preserve common support, we exclude cases in the extreme tail of the distribution (top 5% of hospital stay nights). Specifically, this corresponds to hospital stays longer than 5 days and more than two newly reported chronic conditions across two consecutive waves. These observations likely reflect catastrophic morbidity that may have larger impacts on labour market outcomes. This likely biases our estimates toward zero, so the estimated results should be read as a conservative effect for typical incident shocks. The working sample is conditional on a working status in the last wave allowing us to investigate the effect of a health shock on the hazard of leaving the job market. Eventually, our working sample consists of 4,160 year-to-person observations (1,885 people, 837 males and 1,048 females) that have complete key variables.

In this paper, we consider two binary indicators of incident health shocks: hospitalisation and the new diagnosis of a chronic condition (e.g., heart attack, stroke, asthma, arthritis). The indicator for hospitalisation is constructed based on two questions asking the times of hospital nights and the times of visiting Hospital Emergency Department (ED) over the past 12 months^7^. As to chronic diseases, respondents are asked about the current status of a specific disease condition compared to since the last survey, allowing us to track the change of a specific chronic condition across waves^8^. Noticeably, we are trying to capture the first onset of hospitalisation^9^ or the diagnosis of disease during the survey time, i.e., the occurrence of a health event is conditional on an absent record since the beginning of the survey in 2009 (excluding those who reported hospitalisation/chronic condition at the baseline wave). This measure helps us capture people’s work adjustment to a short-term health shock that is relatively less expected if they have hardly experience up to the point.

In general, there are 1,384 cases (1,047 people) of health events, approximately one-third of our working sample. The proportion experiencing a health event is higher among females (see Table A1) and such a gender difference is statistically significant. In our sample, 375 cases of hospitalisation take around 10% of the working sample; their average number of annual inpatient nights is 0.64, and the average annual number of ED visits is 0.89. There are 1,148 cases identified as the first occurrence of a new chronic condition^10^, and 84% among these are only one new condition: 451 cases are only one new heart disease, and 613 cases are related to only one new other chronic condition. There are 50 cases of an acute shock condition^11^ such as heart attack, stroke or cancer.

As for outcomes variables, labour market participation is identified by self-report on whether they have done any paid work in recent weeks. An active working status also includes those who have done paid work in the last week although they describe their employment status as either retired, unemployed, looking after home or other rather than being employed or self-employed. Other labour market outcomes include weekly working hours, annual working weeks and wages. As shown in Tables 1 and 84.4% of people remain active in the labour market before age 66, and those having experienced health events have a lower likelihood of around 81%. Among the active group of our working sample of 1,566 people, there is only a small proportion with a casual working status and over 70% were working over 30 h per week on a permanent job contract.

**Table 1:** Descriptive statistics

	All		Male	Female	Hospitalisation	New Disease
	Mean	S.D	Mean	Mean	Mean	Mean
*Labour Market Characteristics*						
Working	0.844	0.363	0.859	0.833	0.821	0.810
Annual Wage	28565.0	28297.7	35824.7	23326.1	27148.9	26158.0
Hourly wage	30.36	64.14	30.60	30.19	32.71	30.53
Weekly working hours	31.19	15.84	38.11	26.08	30.54	29.49
Working weeks	37.17	18.47	38.46	36.22	35.45	35.49
Public sector	0.375	0.484	0.278	0.448	0.363	0.377
Self-employed	0.105	0.307	0.185	0.045	0.107	0.085
Permanent contract	0.745	0.436	0.715	0.767	0.747	0.747
*Demographic and SEC characteristics*						
Age	59.26	3.437	59.36	59.18	59.12	59.50
Household size	2.63	1.27	2.79	2.52	2.78	2.54
Married	0.744	0.437	0.795	0.705	0.773	0.731
Education: primary/none	0.113	0.317	0.135	0.097	0.160	0.130
Education: third level/higher	0.452	0.498	0.396	0.495	0.427	0.435
Live in Dublin	0.243	0.429	0.245	0.241	0.243	0.247
Gross household asset	435661.8	613802.6	467537.8	411289.0	446067.4	389391.6
*Social Welfare Entitlements*						
Occupational pension	0.491	0.500	0.522	0.468	0.480	0.487
Private pension	0.121	0.327	0.158	0.094	0.133	0.115
Medical/GP card (only)	0.145	0.353	0.128	0.159	0.171	0.176
Health insurance (only)	0.616	0.486	0.627	0.608	0.603	0.589
Medical/GP card and health insurance	0.052	0.222	0.036	0.064	0.061	0.065
*Health-related Characteristics*						
Number of chronic diseases	1.300	1.182	1.175	1.393	1.424	2.155
Number of physical limitations	1.268	1.561	1.056	1.428	1.483	1.620
Mental health (excellent)	0.224	0.417	0.23	0.219	0.235	0.170
Self-assessed health (excellent)	0.197	0.398	0.162	0.223	0.155	0.122
GP visits	2.655	2.857	2.431	2.823	3.725	3.534
Hospital outpatient visits	0.956	2.741	0.809	1.066	2.501	1.564
Hospital inpatient nights	0.097	0.476	0.088	0.103	0.644	0.152
Hospital Emergency Department visits	0.133	0.398	0.117	0.145	0.885	0.182
Out-of-pocket expenses	269.8	671.6	230.7	299.1	286.8	291.7
Never smoked	0.475	0.499	0.434	0.513	0.444	0.448
*N*	4160		1782	2378	375	1148

The sample includes 1,885 people (837 men, 1,048 women) aged 50–65, observed in at least two consecutive waves between 2009 and 2018. A hospitalisation event is defined as a hospital visit (Emergency Department, or overnight stay) in the past 12 months, conditional on no hospitalisation/ED visit reported since 2009. A new chronic disease is defined as the first onset of a chronic condition during the survey period

Observed confounders in our empirical equation include demographic features, educational qualification, household characteristics, socioeconomic class, and controls for job characteristics (working sector, working hours, contract type), social welfare entitlements (pension type and healthcare entitlements). We utilise a rich set of objective measures of health condition covering the number of chronic diseases, physical limitations, the depression score measured by the Center for Epidemiological Studies-Depression (CES-D), the number of GP visits and hospital outpatients visits over the last 12 months, and smoking behaviours. All health and job-related variables are taken from the last survey period before the occurrence of a health shock.

## Results

### The effect of health shocks on labour market participation (LMP)

**Table 2:** Effects of health events on labour market participation (LMP)

Method	Logit			IPRWA		
	All	Male	Female	All	Male	Female
	(1)	(2)	(3)	(4)	(5)	(6)
*Health event D*:						
Hospitalisation	0.855	1.047	0.776	−0.022	0.003	−0.039
	(0.129)	(0.292)	(0.143)	(0.020)	(0.028)	(0.028)
N(D = 1)	375	143	232	374	142	232
N(D = 0)	2585	1126	1459	2585	1126	1459
N(Y = 0)	451	184	267	451	184	267
New chronic disease	0.824*	0.886	0.791*	−0.025*	−0.010	−0.031*
	(0.082)	(0.155)	(0.100)	(0.014)	(0.021)	(0.018)
N(D = 1)	1148	437	711	1148	436	710
N(D = 0)	3012	1345	1667	3012	1345	1667
N(Y = 0)	648	252	396	648	251	395

* significance at 10% level; ** at 5% level; *** at 1% level. Robust standard errors (in parentheses) are clustered at the household level. Control variables include demographics, education, quantiles of household assets, socioeconomic status, health status and job characteristics measured in the previous wave. Odds ratios are reported for logit models. Labour market participation (LMP) is defined whether reporting employment or self-employment, or reporting non-employment but having done paid work in the past four weeks

Table 2 shows our baseline results from logit regressions and IPRWA that further takes account of the selection problem in a causal identification of the health effect. Gender results are separated given the different labour market patterns and biology-related health conditions. We have restricted our sample to those in an active working status in the last period, so the estimates imply the effect on the hazard of exiting employment. The propensity is estimated by a logistic model. For the hospitalisation analysis, the control group includes respondents with no recorded hospitalisation since baseline up to wave $\:t$. For the chronic-diagnosis analysis, the control group includes respondents who report no new diagnosis of that chronic condition between waves t-1 and t^12^.

**Fig. 1:**
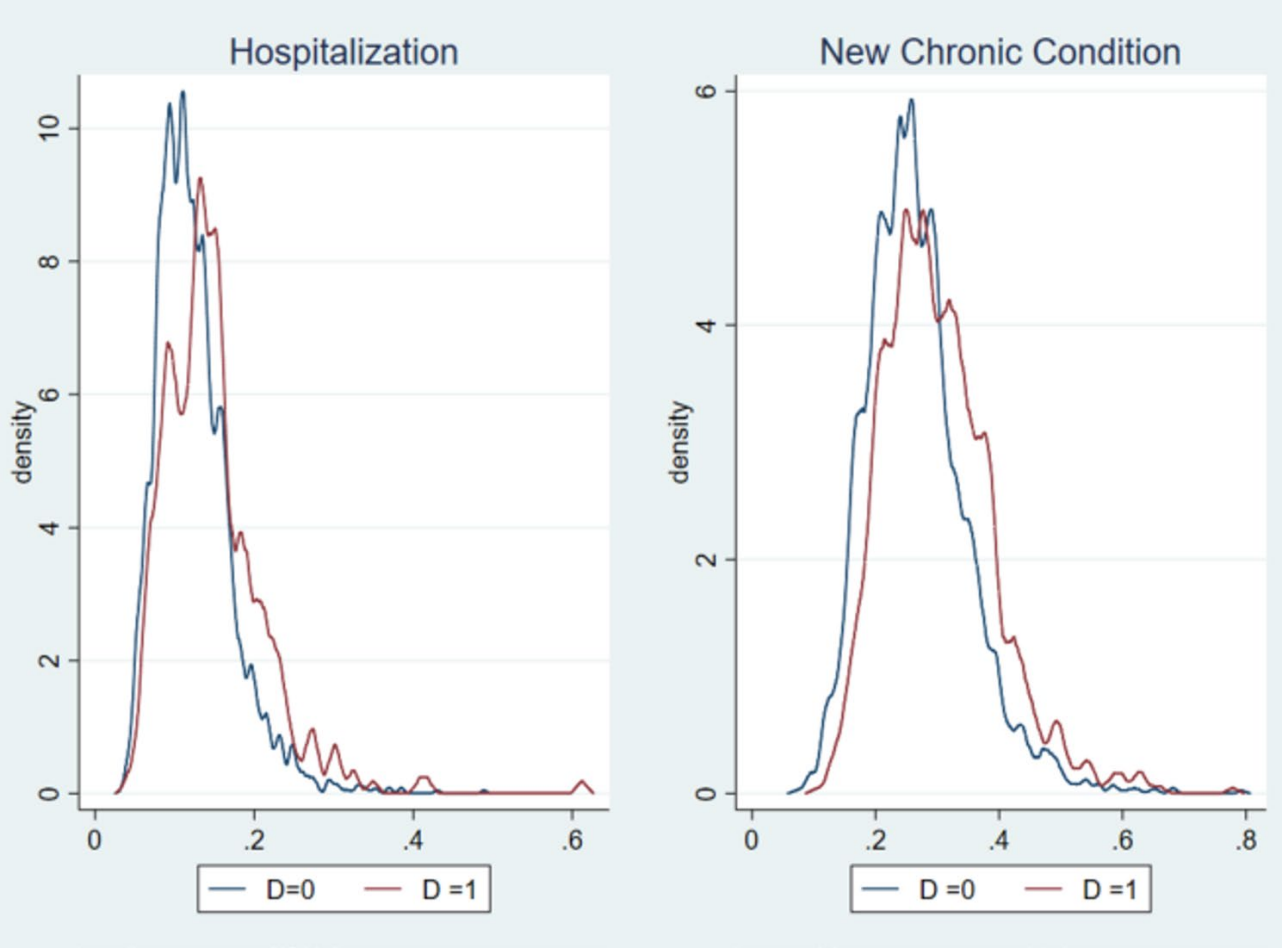
Propensity score plot. Note: For hospitalisation, N=2,960. For the new chronic condition, N=4,160

In the first stage of propensity score estimation, gender and education level are two persistent predictors of a future health event (see Table A3). However, previous health condition is found to be more associated with the emergence of a new chronic condition rather than hospitalisation. Job-related factors, especially the working sector and pension arrangements do not significantly explain differential risks rate of health event identified in our analysis. There are some signs of a correlation between earlier healthcare utilisation conditional on contemporaneous health condition as well, which may be partially related to healthcare accessibility. As shown in Fig. 1, the propensity score largely overlaps among the treated and untreated, and at most only one observation was dropped under the common support restriction.

In the upper panel for hospitalisation, the logit estimate in column (1) shows an odds ratio of 0.855, indicating that the people who experienced a hospitalisation in the past 12 months have 14.5% lower odds of staying in the labour market compared to those without such an event. But the effect is statistically insignificant in all cases and seems to be relatively larger among females. Compared to logit estimation, the estimated ATT from IPRWA is also statistically insignificant, suggesting around 3 percentage lower likelihood of keeping working after a recent hospitalisation. The estimated potential outcome mean of the LMP among the treated group is 0.843, i.e., the counterfactual outcome if the treated group had not experienced a hospitalisation, higher than their true average retention rate of 0.821.

When it comes to a new chronic disease, the estimates are similar in magnitude but become statistically significant at a 10% level. Logit regression shows an odds ratio of 0.824, and IPRWA estimator suggests a weak effect, around two to three% lower likelihood of sustaining a working status among the people who were diagnosed with a specific chronic condition for the first time since the start of the survey year. The effect is also larger among women who have a higher proportion of reporting a new chronic condition relative to men. By severity, the ATT for the small group of acute cases (*n* = 50) is around − 0.274, implying a substantially higher probability of labour market exit by age 65 (approximately a doubling relative to baseline). However, the estimates are subject to small cell size and seem to be disproportionately driven by high SES group. We therefore treat these results as indicative and do not place strong weight on them in the main analysis^13^.

When comparing two types of health events, the estimated odds ratios in two scenarios are both around 0.8, suggesting that the odds of staying active in labour market for people having experienced a health event in the recent 12 months are around 20% lower than that of other who did not. Irrespective of both our health shock variables capturing the first onset of a specific health event, the hospitalisation experience can be more accidental and unpredictable compared to another new health condition, which may be related to a previous health conditions and other individual characteristics. Indeed, across a series of logit estimation, the estimated odds ratio of hospitalisation is less sensitive to different control variables in comparison with that of new diseases going up slightly (see Table A2).

Essentially, no strong effect of health shock on LMP is found from our estimations, and the same applies to other labour market transitions such as partial retirement, a change in working sector or employment contract type (not reported). In total, there are 288 cases of subsequent labour market exit following a health event identified in our sample, representing nearly 20% of all the treated cases. Over half transited from an employment/self-employment status to retirement, 16% became unemployed, and 10% reported a severe sickness condition. Additional regressions also show the significant effect is more around a transition into other non-working status such as looking after home instead of reporting retired or unemployed.

### The effect of health shocks on wage and working time

Having observed a weak effect of a health shock on general labour market activity, we next move to test how some work-related conditions might be affected. The current sample is conditional on a current working status in the present wave. Hence, it is possible that these people have remained in their working capacity after experiencing a health shock earlier.

**Table 3:** *Effects of health events on wages and working time (conditional on working)*

Method	OLS			IPRWA		
	All	Male	Female	All	Male	Female
	(1)	(2)	(3)	(4)	(5)	(6)
*Panel A: Annual wage*						
Hospitalisation	−0.064**	−0.134**	−0.021	−0.065**	−0.129**	−0.019
	(0.031)	(0.065)	(0.033)	(0.032)	(0.063)	(0.037)
New chronic disease	−0.028	0.015	−0.054**	−0.029	0.029	−0.051**
	(0.023)	(0.047)	(0.025)	(0.023)	(0.050)	(0.025)
*Panel B: Hourly wage*						
Hospitalisation	−0.024	0.022	−0.038	−0.025	0.023	−0.030
	(0.036)	(0.073)	(0.040)	(0.037)	(0.070)	(0.043)
New chronic disease	−0.010	−0.014	−0.010	−0.0003	−0.016	0.0003
	(0.038)	(0.074)	(0.044)	(0.043)	(0.094)	(0.995)
*Panel C: Weekly working hours*						
Hospitalisation	0.014	0.025	−0.002	0.017	0.027	−0.004
	(0.031)	(0.053)	(0.037)	(0.031)	(0.055)	(0.038)
New chronic disease	−0.025	−0.037	−0.016	−0.030	−0.039	−0.027
	(0.024)	(0.040)	(0.030)	(0.024)	(0.041)	(0.032)
*Panel D: Total working weeks*						
Hospitalisation	−0.067**	−0.047	−0.077**	−0.069**	−0.049	−0.085**
	(0.029)	(0.046)	(0.038)	(0.030)	(0.048)	(0.040)
New chronic disease	0.007	−0.005	0.007	0.002	−0.008	0.001
	(0.019)	(0.032)	(0.024)	(0.020)	(0.034)	(0.025)

* significance at 10% level; ** at 5% level; *** at 1% level. Robust standard errors (in parentheses) are clustered at the household level. Odds ratios are reported for logit models. All control variables are as in Table 2. The sample is restricted to respondents who remain in work in the current wave following the health event. All outcome variables are standardised. For the hospitalisation analysis, sample sizes for Panels A–D are 2,303, 2,234, 2,406, and 2,406, respectively. For the analysis of new chronic disease, sample sizes for Panels A-D are 3,236, 3,134, 3,377, and 3,374, respectively. Counts of treated and control observations are reported in Table A4. Self-employed respondents are excluded from Panels B-D because TILDA does not collect weekly working hours for this group; excluding them has negligible impact on the Panel A estimates

As shown in the upper two panels of Table 3, we find evidence of a negative effect on annual wages, with patterns differing by gender and event type. Hospitalisation appears to affect men more, reducing annual wages by about 0.13 standard deviation (SD) (approximately 3,600 euros), whereas a new chronic condition reduces women’s annual wages by around 0.05 SD, equivalent to roughly a 6% decline relative to the average annual wages among women in our sample.

However, we find no evidence that these health events affect the hourly wage (annual wages divided by working hours) or weekly working hours which are more directly linked to job productivity. There seems to be a proportional reduction in working time: as shown in the lower two panels of Table 3, we have observed an around 0.07 SD reduction in annual working weeks (about 1.3 weeks), but an insignificant effect on the reported typical weekly working hours after an experience of hospitalisation. The effect of a new chronic condition is smaller and is not statistically significant. The estimates do not show an effect on the calculated hourly wage that partially reflects productivity.

Taken together, these patterns suggest that, conditional on returning to work after a health event, workers are less likely to adjust their routine working capacity in the short run. Many hospitalisations in our data may be acute but short-lived, and any short-run earnings losses are more consistent with temporary absences (e.g., consecutive leave for treatment and recovery) than with persistent reductions in hourly pay or weekly hours. This is also reflected by more pronounced effect on men whose hospitalisations are more likely to be job-related injuries on manual jobs where variable pay is common. By contrast, newly diagnosed chronic conditions seems to be more relevant for the labour market participation decision. Workers who remain employed seem to manage conditions well without a clear loss in productivity. The annual earnings can decline because of changes in job responsibilities rather than change in routine job hours, which may be especially relevant for women in non-manual work.

###  Heterogeneities in labour market outcomes

**Table 4:** Effects of health events on labour market outcomes in subgroups

Subgroups:	Baseline	Non-Dublin	Married	Semi-skilled/unskilled	Public sector	Household asset (lowest 40%)	Education (None/level 1 or 2)
	(1)	(2)	(3)	(4)	(5)	(6)	(7)
*Panel A: Keep working*							
Hospitalisation	−0.022	−0.042*	−0.009	−0.043	−0.060*	−0.934**	−0.035
	(0.020)	(0.236)	(0.023)	(0.047)	(0.036)	(0.041)	(0.028)
N	2959	2243	2197	568	1082	895	1656
New chronic disease	−0.025*	−0.037**	−0.186	−0.070**	0.024	−0.007	−0.018
	(0.014)	(0.018)	(0.018)	(0.036)	(0.025)	(0.028)	(0.021)
N	4160	2242	2198	568	1080	891	1657
*Panel B: Annual wage*							
Hospitalisation	−0.065**	−0.057	−0.072**	−0.027	0.037	−0.035	−0.045
	(0.032)	(0.036)	(0.035)	(0.077)	(0.057)	(0.040)	(0.037)
N	2303	1763	1712	459	857	732	1279
New chronic disease	−0.029	−0.051	−0.023	−0.051	−0.040	−0.029	−0.011
	(0.023)	(0.033)	(0.032)	(0.042)	(0.041)	(0.026)	(0.030)
N	3236	1764	1711	459	865	725	1281
*Panel C: Total working weeks*							
Hospitalisation	−0.069**	−0.090**	−0.067**	−0.150**	−0.071	−0.063	−0.070*
	(0.030)	(0.036)	(0.033)	(0.061)	(0.054)	(0.050)	(0.036)
N	2406	1846	1787	479	890	747	1346
New chronic disease	0.002	−0.024	−0.023	0.037	0.026	0.00002	−0.014
	(0.020)	(0.029)	(0.029)	(0.042)	(0.034)	(0.045)	(0.032)
N	3374	1846	1786	477	900	743	1347

* significance at 10% level; ** at 5% level; *** at 1% level. Estimates are from IPWRA models with the same set of controls as in Table 2. Column (1) provides baseline estimates for the full sample. In Panel A, outcome variable is labour market participation, defined as reporting employment or self-employment, or reporting non-employment but having done any paid work in the past four weeks. In Panels B and C, the sample is restricted to respondents who remain in work in the current wave following the health event. Annual earnings and working weeks are standardised

Our main estimations have established the results about the average treatment effects of a health shock on LMP and work-related outcomes. We also investigate potential heterogeneities in the treatment effects in subgroups with underlying different baseline risks of health shocks, job securities or living conditions. Several patterns appear in our subgroup analysis in Table 4 when compared to the baseline estimates. The effect of a health event on general LMP seems to be higher around the groups living in non-Dublin areas, or semi-skilled/unskilled workers, or the people with the lowest 40% assets. Their adjustments of working time or wages, however, are less clear, which is probably attributable to the relatively lower rate of job survival. Public sector workers are largely unaffected in wage and working time if they continue working after a health event while they are more likely to quit working following hospitalisation. In terms of household factors, the estimated effect is close to the average among married people living with a spouse; no severe effect is observed among the people living alone.

### Further effect of health shocks and the role of healthcare entitlements

As mentioned in the earlier section, our main results largely refer to a short-run effect within no more than two years during which time the institutional rigidities might prevent people from corresponding adjustments. Estimating long-term effects is constrained by data availability and sample attrition. As a result, we attempt to conduct additional regressions on a variety of future results after another two years recorded in the next wave as presented in Table 5 to gauge a potential medium-term effect, which also helps generate insights into the mechanisms.

**Table 5:** Effects of health events on other outcomes in the medium term

	Hospitalisation	New Chronic Disease
	(1)	(2)
Keep working	−0.016	−0.028
	(0.029)	(0.019)
N	2097	2805
Annual wage	0.024	−0.027
	(0.102)	(0.045)
N	1609	2133
Working weeks	−0.029	−0.036
	(0.072)	(0.047)
N	1681	2221
Weekly working hours	−0.033	−0.058
	(0.053)	(0.036)
N	1685	2229
Total household income	−0.011	0.031
	(0.059)	(0.041)
N	1390	1852
GP visits	0.324***	0.232***
	(0.089)	(0.051)
N	1716	2269
Out-of-pocket expenses	0.144	0.025
	(0.096)	(0.045)
N	1720	2274
The number of impairments	0.105	0.151**
	(0.094)	(0.064)
N	2097	2805
The number of chronic diseases	0.091	0.679***
	(0.065)	(0.048)
N	2097	2805
Depression score (CES-D)	−0.014	0.071**
	(0.045)	(0.032)
N	1718	2272

* significance at 10% level; ** at 5% level; *** at 1% level. Standard errors (in parenthesis) are clustered at the household level. Estimates are from IPWRA models with the same set of controls as in Table 2. Working time and wage regressions are conditional on remaining employed in the current wave. All outcome variables are standardised expect general working status and the number of impairments/chronic diseases. Outcomes are measured at the next survey wave, approximately 2–3 years after the health event

Concerning labour market outcomes, the effect of hospitalisation likely diminishes with time, but not in the case of chronic diseases, which can be explained by the likely associations between many diseases in the subsequent period. In our working sample, near one-third people have a consecutive first-time diagnosis of another disease, and there is some evidence on the health deterioration as suggested by the increase in the number of health impairments, chronic conditions, and depression symptoms measured by CES-D score. In relation to potential follow-up treatment, we find a lasting effect on GP visits in both types of health event, suggesting an increase between 0.2 and 0.3 SD in the number of GP visits after another two years, approximately 0.6 to 0.9 more visit in a year in our sample. Albeit an observed effect on health-related conditions, we, again, do not find a strong effect on financial outcomes such as household income and some healthcare expense. Ultimately, these results confirm a negative effect on general health conditions, but these shocks may not necessarily lead to worse economic situations.

Following the results of a consistently positive effect on primary healthcare utilisation, we further explore whether different healthcare entitlements contribute to a heterogenous adjustment to a health event by running regressions that incorporate interaction terms of health event and different healthcare entitlements, taking account of a specific feature of the Irish system in a European context. The Irish health system is largely financed through general taxation. The health spending per capita in Ireland is around one-fifth above the EU average [53]. As a high-income country in Europe, Ireland is the only Western European country that does not provide universal coverage of primary care. The eligibility of public healthcare entitlements varies by age, residency and socioeconomic condition. Residents with low income below a defined threshold or with specific medical conditions are eligible for a Medical Card, which provides access to primary care and hospital services free of charge and medicines with limited co-payments. In 2005, a GP visit card was introduced, and it covers GP charges but not medicines or hospital fees. For the remaining population, the cost of GP services is funded by themselves. Under the two-tier system, many individuals purchase private health insurance (PHI), which mainly provides cover for private or semi-private acute hospital services, and, in some cases, partial reimbursement of certain ambulatory care expenses (e.g. GP visits, routine dental care, physiotherapy etc.).

**Table 6:** The role of healthcare entitlements

Outcome:	Work	Annual Wage	Working Weeks	Working Hours	GP visits	Out-of-pocket expenses
	(1)	(2)	(3)	(4)	(5)	(6)
*Panel A: Hospitalisation*						
D* Medical card/GP card	−0.533	−0.055	−0.241*	−0.111	0.387	−0.193
	(0.608)	(0.085)	(0.125)	(0.119)	(0.285)	(0.430)
D* Private health insurance	0.066	−0.055	−0.080	0.076	0.055	−0.508
	(0.566)	(0.067)	(0.055)	(0.072)	(0.189)	(0.370)
D* Dual coverage	−1.210	0.062	−0.393**	0.157	0.264	−0.537
	(0.779)	(0.104)	(0.198)	(0.108)	(0.334)	(0.425)
*Panel B: New chronic disease*						
D* Medical card/GP card	−0.615*	0.128**	0.007	0.200*	0.184	0.133
	(0.369)	(0.056)	(0.067)	(0.080)	(0.175)	(0.137)
D* Private health insurance	−0.354	0.010	−0.065	0.017	0.018	0.038
	(0.326)	(0.053)	(0.042)	(0.055)	(0.112)	(0.102)
D* Dual coverage	0.279	0.140	−0.340***	0.110	−0.182	0.058
	(0.479)	(0.090)	(0.124)	(0.099)	(0.226)	(0.209)

* significance at 10% level; ** at 5% level; *** at 1% level. Standard errors (in parentheses) are clustered at the household level. All control variables are same as in Table 2. Working-time and wage regressions are conditional on remaining in work in the current wave. GP visits and out-of-pocket (OOP) expenditures are measured at the next survey wave. For the logit model of working status, the raw coefficient is reported. All outcome variables are standardised except working status

Table 6 summarizes related results regarding the potentially heterogeneous effect of four mutually exclusive categories of healthcare entitlements: Medical Card/GP Visit Card holders without PHI (14.5% of our sample), Medical Card/GP Visit Card holders with PHI (5.2%), covered by PHI only (61.6%), and those uncovered by either (18.7%). Compared to the base group without any additional healthcare scheme or coverage, the negative effects of a health event on general labour market participation are relatively larger in the group of 482 people covered by medical card or GP card, in line with some of earlier results on the greater vulnerability of the low SES groups. On average, there is a higher proportion of identified new chronic condition (33.3%) in this group compared to the remaining population with an average of 26.6%, but the difference is statistically insignificant for a hospitalisation event. Nonetheless, once they retain an active working status after a health shock, they seem not to be more vulnerable to a greater wage reduction, and there are even some signs of increased total wage in some groups. A greater reduction of working weeks emerges following a hospitalisation event. This might be partially related to greater usage of primary care services such as GP (4.27 visits) compared to other groups (2.99 visits), but our statistical evidence is suggestive due to relatively small cell size in each category. Our data also shows that the covered people have one more weekly working hour (28.1) compared to the similar covered people without any health shock (27.1). A possible interpretation is that those with free access to primary care gain some protection from this and are better able to resume work given better management of their continuing health condition.

## Robustness checks

Our main identification is on the ground of two main assumptions, the overlap assumption and the conditional independence assumption (CIA). Among all our estimations, at most three observations were dropped under overlap assumption, and the graphical display of estimated propensity score (Fig. 1) also shows support for appropriate overlaps of propensities among the treated and control groups. To further test the success of confounder adjustment in our propensity-based methods, we implement an overidentification test [54] on the covariates and cannot reject the null hypothesis of balanced covariates. In addition, we re-estimate our models based on further trimmed samples to rule out the potential effect of some outliers. The results still hold when we drop a specified proportion of the treatment observations at which the propensity density of the control observations is the lowest. For instance, the estimated ATT of hospitalisation on a general working status is −0.044(0.020), and − 0.029 (0.014) in the case of a new chronic condition in a shrank sample (2,923 for the hospitalisation sample, 4,046 for the chronic disease sample) after trimming 10% of the treated observations. Results are consistently held in estimating wage and working time.

**Table 7:** Oster tests on health-event variables

	Estimated Coefficients	*R* ^2^	Critical$\:{\updelta\:}$
Panel A: Keep working			
New chronic disease	−0.026** (0.013)	0.082	3.59
*Panel B: Wage*			
Hospitalisation	−0.064**	0.565	−90.18
	(0.031)		
*Panel C: Working weeks*			
Hospitalisation	−0.067**	0.124	−13.64
	(0.029)		

* significance at 10% level; ** at 5% level; *** at 1% level. Standard errors in brackets are clustered at a household level. The table provides Oster test results on health event variables that are statistically significant. The critical$\:{\updelta\:}$in the last column indicates the degree of selection on unobservable relative to observables that would be required to explain away the estimated effect. The estimation is based on a linear regression model

Next, we check whether our results are sensitive to the potential violation of CIA. We first use an Oster test (2019) [55] to check the stability of the coefficients of outcome equations to potential confounding unobservables^14^. In a linear regression framework, Oster test utilises the movement of $\:{R}^{2}$ when a different set of controls included informing the relative importance of unobservables to observed controls. In Table 7, we present results based on linear estimations of our outcome model, and the critical $\:{\updelta\:}$ in the last column shows the critical value of proportional selection such that the effect of a specific variable is equal to zero. A value of $\:\delta\:$=1 represents the observables are at least as important as the unobservable, and a value greater than one or below zero lends support to the robustness of the coefficients to omitted variables. For the probability of remaining employed (Panel A), the critical $\:{\updelta\:}$ of the new chronic disease is 3.59, implying that unobserved selection would need to be more than three times as strong as selection on observables to eliminate the estimate. For wage and working-weeks outcomes (Panels B and C), the negative $\:{\updelta\:}$ values smaller than − 1 indicate that coefficient stability can be established in these specifications. Essentially, we do not consider a manifest consequence of unobservables driving main results, given the rich set of our controls in our analysis.

Moreover, a simulation method [56, 57] is applied to check the sensitivity of estimated treatment effects to unobserved confounder in the alternative matching method. We select several observed variables that the potential confounders may behave like such as age, health, education and healthcare entitlement. When the confounder is calibrated to mimic their distributions, the estimated effects are generally stable. Essentially, PSM gives similar ATT in magnitude in most cases but a larger standard error compared to IPRWA estimates.

**Table 8:** Sensitivity checks by health-condition subsamples

Excluding:	Has disease/impairment		Has limiting condition		Chronic conditions > 1	
*Method*	OLS/logit	IPRWA	OLS/logit	IPRWA	OLS/logit	IPRWA
	(1)	(2)	(3)	(4)	(5)	(6)
*Panel A: Keep working*						
Hospitalisation	0.939	−0.007	1.074	0.011	0.827	−0.022
	(0.211)	(0.025)	(0.217)	(0.021)	(0.171)	(0.025)
N	1787	1786	2370	2369	1975	1975
New chronic disease	0.786*	−0.028	0.824	−0.021	0.619***	−0.053**
	(0.112)	(0.018)	(0.102)	(0.015)	(0.096)	(0.022)
N	2354	2353	3207	3207	2605	2602
*Panel B: Annual wage*						
Hospitalisation	−0.083*	−0.090**	−0.065*	−0.065*	−0.118***	−0.115**
	(0.044)	(0.045)	(0.037)	(0.038)	(0.044)	(0.049)
N	1415	1412	1879	1879	1554	1551
New chronic disease	−0.030	−0.026	−0.013	−0.013	−0.006	−0.018
	(0.031)	(0.031)	(0.028)	(0.028)	(0.039)	(0.039)
N	1857	1856	2563	2563	2067	2066
*Panel C: Total working weeks*						
Hospitalisation	−0.071*	−0.071*	−0.029	−0.028	−0.071**	−0.074**
	(0.038)	(0.040)	(0.031)	(0.032)	(0.035)	(0.035)
N	1468	1468	1961	1960	1622	1622
New chronic disease	−0.004	−0.016	0.041*	0.041*	0.024	0.011
	(0.026)	(0.027)	(0.021)	(0.021)	(0.029)	(0.030)
N	1930	1929	2667	2666	2146	2144

* significance at 10% level; ** at 5% level; *** at 1% level. Standard errors (in parentheses) are clustered at the household level. All the control variables are the same as in Table 2. Wages and working weeks variables are standardised

There might be a concern over whether our main results are largely driven by those identified with severe limiting diseases and health shocks which would lead to over-estimated treatment effects. People might anticipate their future labour market transition based on their current health status. Despite the fact that a rich set of health controls for previous health condition has been included in all our models, we check our results by further ruling out this potential bias. In our sample, the average proportion of having a limiting pain/chronic condition is between 13% and 20% among the treated groups, near twice large compared to the non-treated group. We further restrict our sample according to their different health conditions: no reported disease or impairment in the previous period; no limiting pains/chronic condition; has at most one chronic disease currently. As shown in Table 8, a negative effect of health events is still persistent in some cases among these three relatively healthier subsamples. The identification of the first chronic condition seems to be even more influential in terms of the probability of working as shown in the last two columns: the logit estimate suggests the odds of for experiencing a health event is a nearly 40% lower than that of others; the propensity method shows a 5 pp reduction in the likelihood of leaving the labour market. This might be explained by the inexperienced management in response to their first diagnosed disease.

## Discussion and conclusion

The proportion of older people is increasing rapidly among many developed countries, bringing a policy focus to extend working lives to limit the increase in the burden on social welfare systems. Health can be an important factor in labour market exit. In this paper, we examine the effect of a health event on a range of labour market outcomes for a sample of people under the Irish state pension age of 66. Following previous literature, two types of health shock are identified and investigated: the first onset of any one kind of health condition or hospitalisation experience during the TILDA survey span between 2009 and 2018. A double robust estimator method inverse probability weighting regression adjustment (IPRWA) is implemented to account for selection bias to achieve a causal identification of health effects. In contrast with earlier research, we find only a weak negative effect on labour market outcomes. Our estimates demonstrate around three pp reduction in the probability of remaining in work in the subsequent wave following a health event, corresponding to an approximately 16% increase in the baseline exit ratio. In terms of other job-related outcomes, we also do not find a significant detrimental effect on wages or working time except for some negative effects of hospitalisation on annual wages (a reduction by approximately 3,600 euros) and working weeks (1.3 weeks). The effect varies by gender and the shock type and is relatively larger among females and low SES groups. There is no strong evidence of a worsened financial situation such as working wages and household income after a health event. We further investigate underlying mechanisms by exploring the potential heterogeneous effect of health event by different healthcare entitlements in Ireland with consideration for the follow-up treatment and financial constraint. These low SES group people with public healthcare entitlements are more vulnerable to leaving a job on average, however, there is some suggestive evidence on the protective effect on working capacity if they sustain a working status after a shock.

Our findings should be interpreted considering several limitations. First, sample attrition may be non-random: health shocks can trigger drop-out, institutionalisation, or proxy responses, which would tend to attenuate adverse effects if sicker individuals are disproportionately lost^15^. Second, our incident-event measures capture onsets but not severity, duration, or treatment intensity, limiting further discussion about dose-response or more catastrophic events. Third, detection/diagnosis bias is possible: employed or higher SES individuals may be more likely to be screened and diagnosed, which can bias diagnosis effects toward zero by classifying relatively milder cases as treated. Admission thresholds and practice variation can also add noise to the hospitalization measure, again likely diluting true effects. Fourth, our estimates average across recession and recovery years and do not test for a structural break in the relationship between health shocks and labour outcomes. The Great Recession may have permanently shifted employer screening, sick-leave practices, so post-crisis effects could differ from earlier cohorts. Taken together, our results should be interpreted as conservative lower bounds.

Compared to relevant literature, we have found a much smaller effect than the PSM estimate of around 10–16 pp in the work by Garcia Gomez (2011) [15] based on an Irish cohort (1994–2001). The differences should be interpreted cautiously. One plausible explanation is the measurements in health shocks. Garcia Gomez [15] uses a change in SAH (from reporting good/very good to fair/bad/very bad) and a broader question about whether has any chronic physical or mental health problem, illness, or disability. These measures are more likely to capture overall perceived health and functional limitations (e.g., potentially including disability), and may proxy more severe changes in health. People may also report SAH deterioration based on perceived changes in their work capacity. By contrast, we use incident and more time-stamped measures (hospitalization, diagnosis of specific conditions). These are salient but include a range of heath changes that could be acute or clinically manageable. As a result, differences in the underlying constructs of health shock across studies can generate different effect magnitudes even within the same country context.

For another, it is informative to consider policy dimension and demographic changes compared to the earlier cohort, and several reasons might account for a weaker incentive to exit paid work during our sampling period (2009–2018). First, during our study period, there was no statutory sick-leave payment or employment obligation quota of disabled workers in Ireland, and health-related leave terms were largely determined by employer’s policies and employment contract [30]. Compared with many European systems, this limited statutory protection may have increased workers’ reliance on discretionary sick-pay arrangements and state benefits. In the post-crisis environment, companies operated under tight conditions, which likely influenced both leave terms and employees’ decision over illness leave. Second, the receipt of a disability/illness benefits^16^ in Ireland has steadily grown since 2000 but has greatly dropped after 2008 in terms of the total expenditure and volumes [58]. There is also some reduction on the flat rate of related schemes such as the Invalidity Pension^17^. These might weaken the financial incentive correspondingly. Third, the Irish government has endeavoured to create more flexible arrangements for people with disabilities or other illness and published a ten-year strategy for people with disabilities in October 2015^18^. A high retention rate in the workplace in response to a health event might partially stem from these policy orientations. Finally, the dynamic macroeconomic environment might further complicate individuals’ decisions. For example, some people might hold pessimistic anticipation for future economic rebound during the first few years after the crisis; and the positive view could be reinforced and lasting later after the rebound around 2014.

There is only one comparable study by Jones et al. (2020) [29] who find a reduction by 5 pp in the probability of remaining in work following an acute health shock among older workers (age 52–65) in England, approximately 40% increase in the exit risk compared to the baseline exit rate. In our paper, the estimate of acute health shock may be insufficiently representative due to small cell size. Moreover, UK has a different environment with respects to healthcare system and illness/disability policies, which makes it more difficult to make a plausible comparison. Overall, our work contributively adds to the current research by showing the first and updated Irish evidence on the health effect on the labour supply of older workers.

Regarding policy implications, our results speak to systems where primary care is not fully covered, and sick-leave pay is weak or discretionary or the period when related illness/disability benefits were tightened by fiscal constraints. In such environments, health shocks tend to manifest as short absences and income losses rather than cuts to base pay. Policies that improve timely care access and affordability (e.g., lower GP charges, compulsory private health insurance) and that support better return to work (e.g., statutory sick pay, early rehabilitation like Nordics, Germany, France), would contribute to a lower short-term exit rate and mediate the adverse economic and health effects, eventually an important and sustainable strategy for a maintaining robust labour participation in the long term. These considerations could be particularly relevant for European countries with mixed financing system (e.g., Greece), and for middle-income settings with substantial out-of-pocket primary-care costs and labour-market informality (e.g., China). While institutional models are not universal, the core mechanisms including financial protection, timely care, and structured return-to-work are transferable and can be adapted to local capacities.

Future research could extend this work in three directions. First, a valuable next step would be to reconcile our event-based shock measures with SAH and broad chronic-condition/disability measures used by García-Gómez (2011) [15], ideally within a single harmonised health shock measure to provide more direct comparison^19^. Second, longer panels with more frequent observations (or administrative linkages with precise event timing) would enable estimation of medium and long-run dynamics and facilitate complementary designs such as Difference-in-Difference/event-study approaches, including pre-trend assessments and richer treatment-effect trajectories following health shocks. Third, future research could also exploit recent policy changes (e.g., sick leave pay after 2022) in Ireland as quasi-experiments to strengthen causal identification and clarify mechanisms.

## Supplementary Information

Supplementary Material 1.

## Data Availability

TILDA data can be accessed in two ways. Access to all data, including the mortality file, is available only from the TILDA servers at Trinity College Dublin. Application for access can be made via their website: https://tilda.tcd.ie/data/accessing-data/. The publicly accessible dataset files are hosted by the Irish Social Science Data Archive based at University College Dublin, and the Interuniversity Consortium for Political and Social Research (ICPSR) based at the University of Michigan. Researchers wishing to access the data must complete a request form, available on either the ISSDA or ICPSR website (https://www.ucd.ie/issda/data/tilda/).

## Footnotes

1 For example, in the US, the direct cost is an increase in annual OOP expenses equivalent to around 3% of pre-admission annual earnings, but the figure is only 1.5% for European countries and is statistically insignificant for Northern and Southern Europe.

2 Individuals cannot choose to work part-time if they want to be entitled to disability benefits.

3 Trevisan and Zantmio (2016) used the first five SHARE waves and the first six English Longitudinal Study of Ageing (ELSA) waves, spanning from 2002 to 2012. Garcia-Gomez (2011) used ECHPS (1994–2001). The Irish research by Gannon (2005) also used the Irish section from ECHPS (1995–2000). The Irish research by Gannon (2005) also used the Irish section from ECHPS (1995–2000).

4 https://www.cso.ie/en/csolatestnews/pressreleases/2010pressreleases/measuringirelandsprogress2009/.

5 In many OECD countries, employers are responsible for an initial period of paid sick leave [30]. For instance, UK provides around £90/week up to 28 weeks. In Ireland, Sick Leave Act 2022 came into effect in 2023, starting at 3 days per year and 70% of daily pay capped at €110/day. Regarding state benefit, Illness Benefit is a contributory-based weekly payment to people who cannot work because of an illness but only starts from day 6 (if you are off work for more than 5 days) [31].

6 At Wave 1, no participant resided in a nursing home. Our sample excludes participants who were living in a nursing home or residential care at the time of interview. Since we focus on adults aged 50–65 in paid work at baseline, subsequent transitions into nursing homes are rare (in total 1% and the mean age is 83.7 years) [50] and has little impact on main estimates.

7 In the questionnaire, respondents were asked “In the last 12 months, how many times did you visit a hospital Emergency Department (sometimes called A&E or Accident and Emergency) as a patient?” and “In the last 12 months, about how many visits did you make to a hospital as an outpatient? (include all types of consultations, tests, operations, procedures or treatments)”.

8 In the first wave, respondents were asked “Has a doctor ever told you that you have any of the conditions? ” In the following surveys, respondents were asked “Since your last interview, has a doctor ever told you/that you have any of the conditions?” The health conditions include heart diseases (e.g., high blood pressure, angina, heart attack) and other conditions (e.g., asthma, arthritis, osteoporosis, cancer). Details of conditions and the numbers are in Table A1.

9 The survey question asks hospitalisation in the recent one year; therefore, our measure is limited in tracking all hospitalisation during an average two-year interval across waves. But we still restrict our measure to the first reported hospitalisation as the best effort to reduce the effect of prior event experience.

10 Heart diseases include high blood pressure (or hypertension), angina, a heart attack, congestive heart failure, diabetes (or high blood sugar), stroke, ministroke or TIA, high cholesterol, heart murmur, abnormal heart rhythm, atrial fibrillation, abnormal heart rhythm (not atrial fibrillation). Other chronic conditions include asthma, arthritis, osteoporosis, cancer, Parkinson’s diseases, emotional/nervous/psychiatric problems, alcohol abuse, substance abuse, Alzheimer’s disease, dementia, serious memory impairment, stomach ulcer, varicose ulcers, cirrhosis (or serious liver damage), thyroid problems, chronic kidney disease, severe anaemia, epilepsy, chest infection.

11 Following previous literature, acute shocks refer to the incidence of a cancer, stroke or myocardial infarction. These types of health shocks are considered less anticipated in terms of the timing (e.g. [12, 51, 52]).

12 Estimates include cases where respondents report consecutive new chronic diagnoses across waves (of different conditions). All models control for the lagged count of chronic conditions to account for comorbidity dynamics. In robustness checks, we also estimate effects in subsamples without any pre-existing conditions.

13 In our sample, 15 people out of 50 exited labour market after an acute shock, and half of them are from high SES group: 8 of them have a third level or higher education, 5 people were doing at least a managerial or technical job. Hence, we do not emphasize the estimates too much for the sake of sample representativeness. For other types of shocks such as hospitalisation and other new chronic condition, the SES difference is generally similar.

14 In the main outcome models, the coefficients of key control variables (e.g., wage, healthcare plan and health behaviour) are also insensitive to selection on unobservables. We also checked the coefficient stability in our propensity-score (treatment) models, and the results are robust. Detailed results are available upon request.

15 As the currently accessible public TILDA files no longer support reconstruction of the original condition-specific incidence measures and sample pipeline, we provide a proxy sample-flow using public variables (age restriction, consecutive-wave participation, baseline health screens using a long-term-condition indicator and hospital/ED events, and lagged employment). See Appendix Table A5.

16 Disability Allowance is a means-tested weekly payment made to people between the ages of 16 and 66 who, because of a specified disability, are substantially restricted in undertaking work which would otherwise be suitable having regard to the person’s age, experience and qualifications. For the purposes of the scheme, a specified disability is defined in regulations and it must have continued or expected to continue for at least one year. The disability is certified by way of a medical report completed by the recipient’s doctor, which is reviewed by a Medical Assessor from the Department of Social Protection. The maximum weekly rate is €188 and recipients can also claim for an adult dependent (€124.80) or a child dependant (€29.80). Illness Benefit is a short-term contributory-based weekly payment to people who cannot work because of an illness. No payment is made for the first 6 days of illness, and it is now payable for a maximum of 2 years. The disability is certified by way of a weekly medical certificate completed by the recipient’s doctor. The maximum weekly rate is €188, and recipients can also claim for an adult dependent (€124.80) i.e. a spouse, civil partner or cohabitant.

17 Invalidity Pension is a contributory-based weekly payment to people who cannot work because of a long-term illness or disability. To qualify the recipient must have been incapable of work for at least 12 months and be likely to be incapable of work for at least another 12 months (or be permanently incapable of work). Like Disability Allowance, the disability is certified by way of a medical report completed by the recipient’s doctor, which is reviewed by a Medical Assessor from the Department of Social Protection. Since 2002 the weekly rate has risen by 57% from €123.30 to €209.80 in 2009 after which it was reduced to €193.50 by 2013.

18 Government of Ireland. (2015). Comprehensive Employment Strategy for People with Disabilities 2015–2024.

19 We were unable to replicate the SAH change specification because the currently accessible public TILDA release has limited information in some health variables. Also, the underlying panel has changed due to participant withdrawals.
