# Supplementary material for: Health shocks and labour market outcomes: evidence from The Irish Longitudinal Study on Ageing (TILDA)
Source: Health Econ Rev. 2026 Feb 25;16:42. doi: 10.1186/s13561-026-00742-y (PMC13041207; doi:10.1186/s13561-026-00742-y)
Supplement: Supplementary file 1 — Supplementary Material 1. [file 13561_2026_742_MOESM1_ESM.docx]

## Appendices

Table A1 Summary of health events

|  |  | ***Wave 2***  ***(N=1203)*** | ***Wave 3***  ***(N=1258)*** | ***Wave 4***  ***(N=1003)*** | ***Wave 5***  ***(N=696)*** | ***Total (N=4160)*** |
| --- | --- | --- | --- | --- | --- | --- |
| ***Hospitalization*** |  |  |  |  |  |  |
| Overnight | Male | 14 | 17 | 18 | 14 | 63 |
|  | Female | 37 | 31 | 22 | 12 | 102 |
| Emergency Department visit | Male | 34 | 46 | 22 | 22 | 124 |
|  | Female | 71 | 54 | 43 | 30 | 198 |
| ***New Chronic Disease*** |  |  |  |  |  |  |
| Heart disease | Male | 75 | 77 | 47 | 37 | 236 |
|  | Female | 121 | 87 | 48 | 43 | 299 |
| Other chronic conditions | Male | 53 | 74 | 51 | 54 | 232 |
|  | Female | 116 | 188 | 84 | 77 | 465 |
|  |  |  |  |  |  |  |
| ***Overlapped cases*** | Male | 9 | 23 | 10 | 10 | 52 |
|  | Female | 34 | 28 | 16 | 9 | 87 |

Note: Heart disease (based on PH201) includes high blood pressure (or hypertension), angina, a heart attack, congestive heart failure, diabetes (or high blood sugar), stroke, ministroke or TIA, high cholesterol, heart murmur, abnormal heart rhythm, atrial fibrillation, and abnormal heart rhythm (non-atrial fibrillation).

Other chronic conditions (based on PH301) include asthma, arthritis, osteoporosis, cancer, Parkinson’s diseases, emotional/nervous/psychiatric problems, alcohol abuse, substance abuse, Alzheimer’s disease, dementia, serious memory impairment, stomach ulcer, varicose ulcers, cirrhosis (or serious liver damage), thyroid problems, chronic kidney disease, severe anaemia, epilepsy, and chest infection.

Table A2 Logit estimation of the effect of health events on labour market participation (LMP)

| Specification | (1) | (2) | (3) | (4) | (5) | (6) |
| --- | --- | --- | --- | --- | --- | --- |
| *Health events D:* |  |  |  |  |  |  |
| ***Hospitalization*** | 0.800 | 0.822 | 0.809 | 0.834 | 0.852 | 0.855 |
|  | (0.118) | (0.122) | (0.120) | (0.124) | (0.128) | (0.129) |
|  |  |  |  |  |  |  |
| ***New Chronic Disease*** | 0.710*** | 0.767*** | 0.769*** | 0.813** | 0.825* | 0.824* |
|  | (0.066) | (0.074) | (0.074) | (0.080) | (0.082) | (0.082) |
|  |  |  |  |  |  |  |
| *Covariates* |  |  |  |  |  |  |
| Demographic characteristics |  | YES | YES | YES | YES | YES |
| Social economic status |  |  | YES | YES | YES | YES |
| Social welfare entitlements |  |  |  | YES | YES | YES |
| Health status (t-1) |  |  |  |  | YES | YES |
| Job characteristics (t-1) |  |  |  |  |  | YES |

Note: * significance at 10% level; ** at 5% level; *** at 1% level. Standard errors (in brackets) are clustered at the household level. The sample sizes are 2960 and 4160 for the hospitalization and chronic disease analyses, respectively. All regressions include wave fixed effects. Odds ratios are reported. Health status (t-1, measured in the previous wave) includes the number of impairments, the number of diseases, the depression score (CES-D), the number of GP visits in the past 12 months, and smoking behaviour. Job characteristics (t-1, measured in the previous wave) include sector, contract type, annual working time, and wage. In column (6), missing job characteristics are imputed using predicted values from separate OLS regressions; an indicator for imputed observations is included.

Table A3 Logit estimation of the propensity score for health events

|  | **Hospitalization** | | **New Chronic Disease** | |
| --- | --- | --- | --- | --- |
|  | Odds Ratio | Std. Err. | Odds Ratio | Std. Err. |
| Age | 0.771 | 0.407 | 1.153 | 0.394 |
| $\mathrm{Age}^{2}/100$ | 1.260 | 0.562 | 0.914 | 0.263 |
| Male | 0.697** | 0.099 | 0.840* | 0.074 |
| Household size | 1.080* | 0.058 | 0.943 | 0.033 |
| Household asset (lowest 20%) | 0.938 | 0.211 | 1.111 | 0.149 |
| Household asset (20-40%) | 0.900 | 0.178 | 1.037 | 0.124 |
| Household asset (40-60%) | 1.155 | 0.220 | 0.982 | 0.117 |
| Household asset (60-80%) | 1.137 | 0.218 | 1.116 | 0.131 |
| Household asset (top 20%) | 1.343 | 0.270 | 0.988 | 0.127 |
| Married, living with spouse | 1.140 | 0.197 | 1.103 | 0.116 |
| Widowed | 0.834 | 0.257 | 0.888 | 0.165 |
| Education: secondary | 0.633** | 0.118 | 0.873 | 0.107 |
| Education: third/higher | 0.653** | 0.138 | 0.734** | 0.100 |
| Live in Dublin | 0.771* | 0.124 | 1.054 | 0.107 |
| Live in a rural area | 0.764* | 0.107 | 1.009 | 0.089 |
| Occupational pension | 1.125 | 0.159 | 1.058 | 0.093 |
| Private pension | 1.266 | 0.222 | 1.052 | 0.120 |
| Medical/GP card (only) | 1.299 | 0.265 | 1.315** | 0.172 |
| Both medical/GP card/insurance | 1.209** | 0.205 | 1.057 | 0.110 |
| Health insurance (only) | 1.813 | 0.517 | 1.421** | 0.244 |
| # Of impairments (t-1) | 1.007 | 0.041 | 1.105*** | 0.027 |
| # Of chronic disease (t-1) | 1.072 | 0.062 | 0.906*** | 0.032 |
| Never smoked (t-1) | 0.786** | 0.094 | 0.925 | 0.068 |
| CES-D score (t-1) | 1.074 | 0.063 | 1.082** | 0.039 |
| GP visits (t-1) | 1.014 | 0.070 | 1.168*** | 0.046 |
| Hospital outpatient visits (t-1) | 1.232** | 0.100 | 0.949 | 0.036 |
| Public sector (t-1) | 0.968 | 0.128 | 0.933 | 0.078 |
| Self-employed sector (t-1) | 0.698 | 0.191 | 0.920 | 0.154 |
| Permanent contract (t-1) | 0.889 | 0.149 | 1.197* | 0.130 |
| Total working hours (t-1) | 1.063 | 0.077 | 0.934 | 0.041 |
| Wage (t-1) | 0.970 | 0.072 | 1.003 | 0.042 |
| Professional | 0.520 | 0.235 | 0.901 | 0.237 |
| Managerial/technical | 0.702 | 0.243 | 0.865 | 0.192 |
| Non-manual | 0.606 | 0.206 | 0.668* | 0.147 |
| Skilled | 0.939 | 0.342 | 0.699 | 0.164 |
| Semi-skilled | 0.755 | 0.266 | 0.626** | 0.144 |
| Unskilled | 1.024 | 0.472 | 0.632 | 0.197 |

Note: * significance at 10% level; ** at 5% level; *** at 1% level. Standard errors are clustered at the household level. The following variables are standardised: depression score, GP visits, hospital outpatient visits, working hours, and wage. For presentation, coefficients on wave indicators and the imputation-flag flag variable are not reported. The reference category for household assets is the missing group.

|  | *OLS* | | | *IPRWA* | | |
| --- | --- | --- | --- | --- | --- | --- |
|  | Grouped | Male | Female | Grouped | Male | Female |
| *Panel A: Annual Wage* | | | | | | |
| Hospitalization |  |  |  |  |  |  |
| N(D=1) | 288 | 111 | 177 | 287 | 109 | 177 |
| N(D=0) | 2015 | 858 | 1157 | 2015 | 858 | 1157 |
| New Chronic Disease |  |  |  |  |  |  |
| N(D=1) | 865 | 327 | 538 | 865 | 325 | 537 |
| N(D=0) | 2371 | 1049 | 1322 | 2371 | 1049 | 1322 |
| *Panel B: Hourly Wage* | | | | | | |
| Hospitalization |  |  |  |  |  |  |
| N(D=1) | 283 | 110 | 173 | 282 | 108 | 173 |
| N(D=0) | 1951 | 818 | 1133 | 1951 | 818 | 1133 |
| New Chronic Disease |  |  |  |  |  |  |
| N(D=1) | 848 | 317 | 531 | 848 | 316 | 530 |
| N(D=0) | 2286 | 1000 | 1286 | 2286 | 1000 | 1286 |
| *Panel C: Weekly Working Hour* | | | | | | |
| Hospitalization |  |  |  |  |  |  |
| N(D=1) | 298 | 119 | 179 | 297 | 116 | 179 |
| N(D=0) | 2108 | 911 | 1197 | 2108 | 911 | 1197 |
| New Chronic Disease |  |  |  |  |  |  |
| N(D=1) | 901 | 345 | 556 | 901 | 343 | 555 |
| N(D=0) | 2476 | 1113 | 1363 | 2476 | 1113 | 1363 |
| *Panel D: Annual Working Weeks* | | | | | | |
| Hospitalization |  |  |  |  |  |  |
| N(D=1) | 297 | 119 | 178 | 296 | 116 | 178 |
| N(D=0) | 2109 | 911 | 1198 | 2109 | 911 | 1198 |
| New Chronic Disease |  |  |  |  |  |  |
| N(D=1) | 900 | 343 | 557 | 900 | 341 | 530 |
| N(D=0) | 2474 | 1115 | 1359 | 2474 | 1115 | 1286 |

Table A4 The number of treated and controls

Table A5 Proxy sample flow using public-release variables

| Steps | Restrictions | Individuals |
| --- | --- | --- |
| **1** | Observed at least in one wave | 8,501 |
| **2** | Age 50-65 (in all waves) | 5,253 |
| **3** | Observed in at least 2 consecutive waves | 4,707 |
| 4a | No long-term condition at the first observed wave | 3,075 |
| 4b | No reported hospitalisation at the first observed wave | 3,087 |
| **4c** | No both long-term condition and hospitalisation at first observed wave | 4,254 |
| **5** | Observed employed status in the previous wave | 2,538 |

Note: This table reports a proxy sample selection flow based on the variables available in the current public TILDA release. Our working sample includes age 50-65, presence in at least two consecutive waves, no a long-term condition or a hospital/ED event indicator measured at the respondent’s first observed wave, and a lagged employment restriction (employed in the previous wave). These counts are intended to document sample restrictions and potential attrition under the publicly accessible measures; they do not exactly replicate the original condition-specific incidence construction used in the main analysis, which required variables that are no longer consistently available across waves.

Figure A1 Propensity score plot (wage sample)


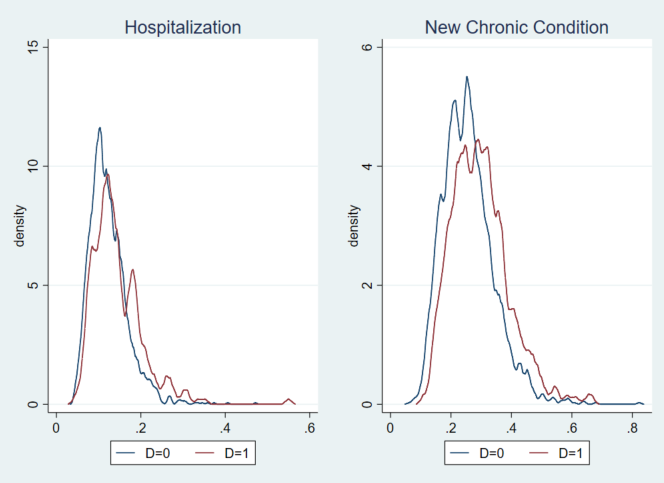


Note: For hospitalization, N=2509. For new chronic condition, N=3512
